# Sociodemographic and gynaecological factors that influence uptake of cervical cancer screening. A cross-sectional study in Calabar, Nigeria

**DOI:** 10.4314/ahs.v22i4.13

**Published:** 2022-12

**Authors:** Boniface U Ago, Efiok E Efiok, Sunday E Abeng

**Affiliations:** 1 Department of Obstetrics and Gynaecology, University of Calabar, Nigeria; 2 Department of Obstetrics and Gynaecology, University of Calabar Teaching Hospital, Nigeria

**Keywords:** Cervical cancer screening uptake, risk factors

## Abstract

**Background:**

Voluntary screening for cervical cancer has not been very effective in sub-Saharan Africa. Awareness and presence of risk factors may drive the need to screen.

**Objective:**

To characterise sociodemographic and gynaecological factors as promoters of screening uptake.

**Methodology:**

The setting was a women health rally in Calabar, Nigeria with women from different towns/ villages in Cross River State. An interviewer-administered questionnaire assessed sociodemographic and gynaecological risk factors for cervical cancer, previous Pap smear, and acceptance to screen. Data inputted in EpiInfo 7, and GraphPad Prism 7.04 statistical software's, were analysed using descriptive and inferential statistics.

**Results:**

One hundred and eighty (180) women gave consent for inclusion in the study. The age ranged from 21 to 65 with a mean of 39.8±10.3 years. With 52.22% of respondents accepting and 47.78% declining to screen, test of association showed that knowledge of cervical cancer, history of multiple sexual partners, and presence of offensive watery vaginal discharge significantly reduced the number of women who refused to screen. Previously screened women were not more likely to accept screening.

**Conclusion:**

Screening for cervical cancer was still poor. Cervical cancer knowledge and recognition of risk factors improve screening uptake.

## Introduction

Cervical cancer is a disease that affects the health of the affected women with severe socioeconomic and psychological implications on their families. It is preventable by early detection and prompt treatment as well as by HPV vaccination. Women's knowledge of the disease is likely to increase their propensity to screen.^1^

Cervical cancer is still a problem in sub-Saharan Africa where comprehensive screening for the disease is deficient. The World Health Organization (WHO) has launched a global strategy to accelerate the elimination of cervical cancer as a public health problem.^2^

The annual number of new cases of cervical cancer have been projected to increase from 570,000 to 700,000 between 2018 and 2030, with a projected annual death rising from 311,000 to 400,000.2 However, effective utilization of evidence-based interventions such as cervical cancer screening, HPV vaccination, and management of detected disease, can accelerate the elimination of this disease. The WHO hopes that achieving the 90-70-90 targets by 2030 would avert over 62 million cervical cancer deaths by 2120.^2^

Regular screening can prevent the disease, but receiving encouragement to do screening increases screening uptake by 5.24 times.^3^

Uptake of cervical cancer screening may be affected by some factors such as age, marital status, knowledge, income, and accessibility of screening services.^3^ These sociodemographic factors when targeted in a public health strategy for uptake of cervical cancer screening, surveillance and treatment of early disease, could have significant impact on disease prevention, early diagnosis, as well as prompt and effective treatment.

Women with multiple sexual partners are more likely to acquire sexually transmitted infections. Utilization of cervical cancer screening was found to be 6.9 times higher in a group of commercial sex workers.^4^ The presence of abnormal vaginal discharge may be the driver for the uptake. Targeting abnormal vaginal discharge as a sentinel gynaecological factor for cervical dysplasia may be an option for early diagnosis.

The early stage of the disease is mostly asymptomatic. However, patients may have slight offensive watery vaginal discharge with or without slight postcoital bleeding, which may go unnoticed or misunderstood until the advanced stage of the disease. Proper information about risk factors and early warning signs for the disease may improve screening uptake.

Women knowledge of cervical cancer has been shown to increase uptake of cervical cancer screening.1 Prior counselling by doctors/nurses and knowing someone with cervical cancer significantly increased uptake of Pap smear.^5^ It has also been reported that higher level of education, though significantly associated with increased awareness of Pap smear, was not associated with increased uptake.^6^ In a study in Ibadan, Nigeria, there was a population-based prevalence of 7.6% of epithelial abnormalities.^7^ It is important to increase knowledge of key sociodemographic and gynaecological factors that can drive the need for women to voluntarily accept cervical cancer screening services which enhance early diagnosis and prompt treatment.

This study assessed some sociodemographic and gynaecological characteristics in a cross-section of women and compared them between those who accepted to screen with those who declined. It was important to know if these characteristics influenced women's acceptance to screen.

## Setting, Study Design, And Methodology

This was a questionnaire-based cross-sectional study. The subjects were women drawn from different parts of Cross River State to Calabar for a rally organized by the Medical Women Association of Nigeria (MWAN) to create awareness on women health with special emphasis on cervical cancer. It was a three-day event held at the Women Development Centre in Calabar in April 2017 with over 300 women in attendance. An intention of the rally was to offer an opportunity for Pap smear (cervical cancer screening) to volunteers in an appropriate facility. Health education on cervical cancer was followed by request and counselling of the women to fill a questionnaire for the conduct of this research. It was an interviewer assisted structured questionnaire and only those who consented were included. These researchers were resource persons, and the permission for the study was included in the ethical clearance for the health rally.

The sociodemographic and gynaecological characteristics enquired were age, marital status, menarche, coitarche, parity, and menopause. Risk factors for cervical cancer enquired were offensive watery vaginal discharge, post coital bleeding, history of multiple sexual partners and family history of cervical cancer. We enquired about knowledge of cervical cancer by asking some questions about the disease and the affected part of the body. Other parameters included previous screening for cervical cancer and the acceptance to do Pap smear now.

The study size was calculated using the formula: z^2^p(1-p)/d^2^ where z is the standard normal variant at 5% type 1 error (p=0.05) which is 1.96 and p is the expected proportion in population based on a previous study, and d is the absolute error or precision which is 0.05

The prevalence in a previous study^7^ was 7.6%

The sample size thus was 1.962(0.076*0.924)/0.05^2^ which was 108.

Attrition of 20% (19) gave a sample size of 127. To increase the power further 180 participants were recruited. Data was inputted in the EpiInfo version 7.2.3.1 CDC Atlanta Georgia, USA statistical software. A page for each woman contained her characteristics listed above.

We presented the results in descriptive statistics.

The mean and median ages were calculated from the inputted ages, which are presented in Table 1 as a range. Similarly, the ages at menarche and coitarche inputted individually are shown as a range. Parity was inputted as number of deliveries, but represented here as a range.

Marital status shows the number of women that were married, divorced, single, or widowed and their simple percentages.

Menopause was captured by asking ‘have you stopped menstruating signalling end of reproductive life’ with a ‘yes’ or ‘no’ response.

An enquiry was made about risk factors such as history of multiple sexual partners, and family history of cervical cancer, and factors such as post coital bleeding and offensive watery vaginal discharge that are early signs of cervical cancer. Enquiry was made of knowledge about cervical cancer, previous screening for cervical cancer and willingness to ‘do Pap smear now’. The responses were also ‘yes’ or ‘no’.

The health rally offered an opportunity for the women to either accept or reject screening for cervical cancer at a designated screening facility. A 2x2 contingency table to test for association of characteristics between those who accept to screen and those who decline was done using Fisher's exact test presented by GraphPad Prism 7.04, CA, USA. Unconditional logistic regression of age (range) and coitarche (range) was done using EpiInfo logistic regression model, to test if age and coitarche were significant characteristics between those who accepted to screen and those who declined. Results presented in Figure A, B, C, D.

## Results

Table 1a.

**Table 1a:** Sociodemographic and Gynaecological Characteristics

Age in years	frequency	Percentage	mean	median
20 – 29	26	14.44	39.75±10.34	41
30 – 39	56	31.11		
40 – 49	58	32.22		
50 – 65	40	22.22		
total (n)	180	100.00		
**Menarche**				
11–13	53	29.78		
14 – 17	97	54.49	14.48±2.05	14
≥18	28	15.73		
total (n)	178	100.00		
**Coitarche**				
12 – 14	13	7.34		
15–19	154	87.01	17.28±1.92	18
≥20	10	5.65		
total (n)	177	100.00		
**Parity**				
0	49	27.22	2.29±1.98	
1 – 4	104	57.78		2
≥5	27	15.00		
total (n)	180	100.00		
**Marital status**				
Married	134	74.44		
Divorced	5	2.78		
Single	32	17.78		
Widowed	9	5.00		
total (n)	180	100.00		
**Menopause**				
Yes	51	28.33		
No	129	71.67		
total (n)	180	100.00

**Table 1b:** Logistic regression of age range and coitarche range

Term	Odds Ratio	95%	C.I.	Coefficient	S. E.	Z-Statistic	P-Value
**Age range (30–** **39/20–29)**	1.0862	0.4276	2.7594	0.0827	0.4757	0.1738	0.8620
**Age range (40–** **49/20–29)**	2.0555	0.8039	5.2555	0.7205	0.4790	1.5043	0.1325
**Age range (50–** **59/20–29)**	0.8167	0.2915	2.2876	-0.2025	0.5255	-0.3854	0.7000
**Age range (60–** **69/20–29)**	2.3331	0.3617	15.0513	0.8472	0.9512	0.8907	0.3731
**Coitarche range** **(15–19/12–14)**	0.7697	0.2550	2.3232	-0.2617	0.5636	-0.4643	0.6424
**coitarche range** **(≥20/12–14)**	1.7493	0.3141	9.7434	0.5592	0.8762	0.6382	0.5233

The study population had an age range of 21–65 years with a mean of 39.75±10.34 years and a median of 41 years. The mean and median ages were calculated from the inputted ages, which are presented in Table 1 as a range. Similarly, the ages at menarche and coitarche inputted individually are shown as a range. The mean age at menarche was 14.48±2.05 years, and the median age was 14 years. The mean age at coitarche was 17.28±1.92 years, and the median was 18 years. Parity was inputted as number of deliveries, but represented here as a range. The mean parity was 2.29±1.98, with a median of two. Marital status shows the number of women that were married, divorced, single, or widowed and their simple percentages. Our study populations comprised 74.44% married, 2.78% divorced, 17.78% single, and 5.00% widowed women.

Menopause was captured by asking ‘have you stopped menstruating signalling end of reproductive life’ with a ‘yes’ or ‘no’ response. Menopause had occurred in 28.33% of our responders.

In Table 1b, logistic regression between age ranges, as well as age range at coitarche, did not reveal statistically significant differences.

**Figure 1:**
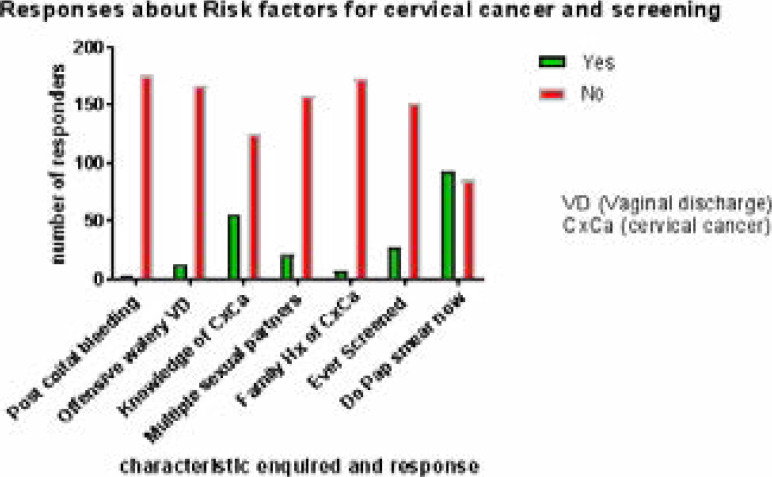
Gynaecological risk factors, Family History (Hx) of cervical cancer, previous screening, and acceptance to screen (Do Pap smear at the rally). A ‘Yes’ response affirmed while a ‘No’ negated the enquired characteristic.

In Figure 1, an enquiry was made about risk factors such as history of multiple sexual partners, and family history of cervical cancer, and factors such as post coital bleeding and offensive watery vaginal discharge that are early signs of cervical cancer. Enquiry was made of knowledge about cervical cancer, previous screening for cervical cancer and willingness to ‘do Pap smear now’. The responses were also ‘yes’ or ‘no’.

Postcoital bleeding was reported in 4 (2.22%) women, while offensive watery vaginal discharge was present in 13 (7.22%) women.

Of the 180 women, 55 (30.56%) had knowledge of cervical cancer, while 22 (12.22%) have had multiple sexual partners (msp) and 7 (3.89%) have had a family member with cervical cancer. Also, 28 (15.56%) of the women had previously done Pap smear. Interestingly, 94 (52.22%) women accepted to screen (do Pap smear now) while 86 (47.78%) rejected the offer.

Figure 2 A, B, C, D

**Figure 2A, B, C, D:**
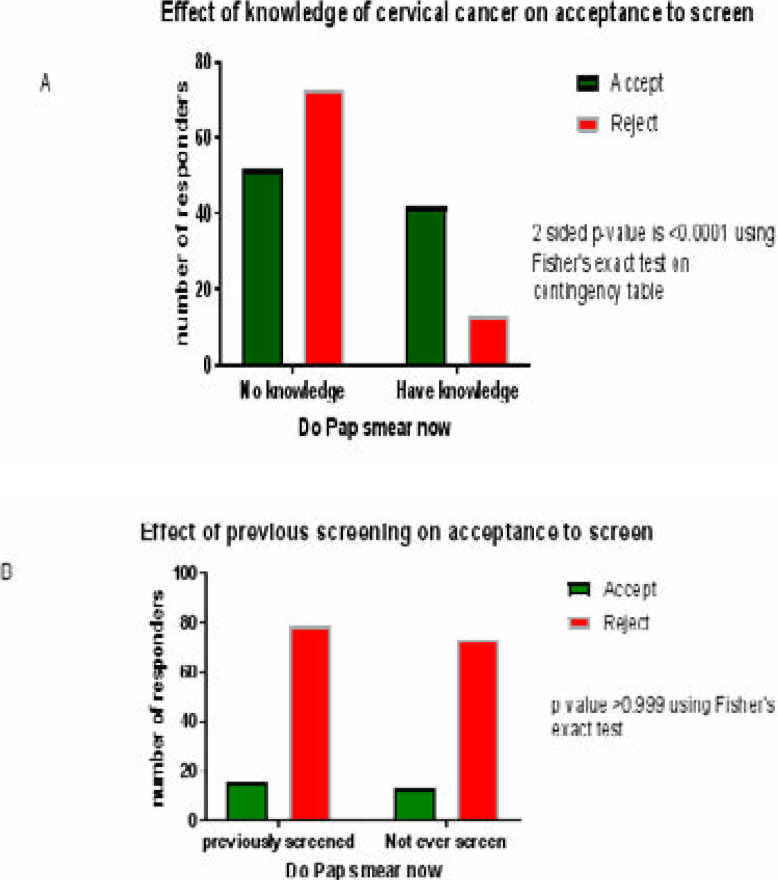

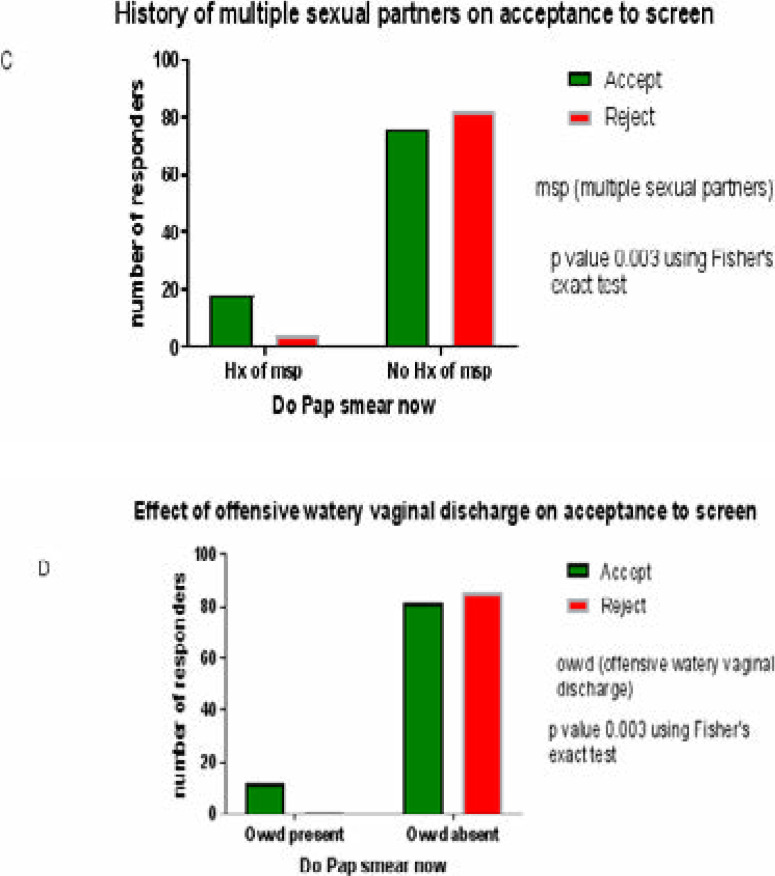
Acceptance to screen for cervical cancer was tested on the bases of knowledge of cervical cancer, previous screening for cervical cancer, History (Hx) of multiple sexual partners, and offensive watery vaginal discharge between those who accepted and those who rejected the characteristic.

With 52.22% accepting and 47.78% rejecting the offer to screen for cervical cancer, the characteristics in Figure 1 were compared in a 2x2 contingency table.

In Figure 2A, knowledge of cervical cancer significantly reduced the number of women who declined to screen (do Pap smear now) compared to those who had no knowledge, p-value <0.0001

In Figure 2B, previous screening for cervical cancer did not significantly affect acceptance or rejection to screen among the women studied, p-value >0.999

Figure 2C, shows that women who have had multiple sexual partners were more likely to screen (do Pap smear now). The number of women who declined to screen was significantly less among those who have had multiple sexual partners than among those who have not, p-value 0.003

Figure 2D, shows that the presence of offensive watery vaginal discharge (owvd) significantly reduced the number of women who rejected the offer to screen when compared to those who had no owvd, p-value 0.003

The p values are shown on the right side of the graph. P value <0.05 is statistically significant.

## Discussion

Voluntary screening programs such as screening for cervical cancer in resource poor countries, have suffered poor uptake probably because the drive or motivation for women to present themselves for screening have not been clearly defined.

The age range of women in our study was from 21 – 65 years, which covers the age for screening for cervical cancer, the same in an earlier study.^3^ The mean age of women in our study was 39.75±10.34 years, which is close to the 38.64±9.39 years in the Jordanian study.^3^

Means of age, age at menarche, and age at coitarche were within previously reported studies. Logistic regression done as shown in Table 1b did not reveal any significant differences. The median parity of the women was 2, and 74.44% of the women were married. Menopause had occurred in 28.33% of the women.

In Figure 1, responses on presence of risk factors such as multiple sex partners, warning signs such as postcoital bleeding and offensive watery vaginal discharge were shown. Having multiple sexual partners was a risk factor for cervical cancer,^8^ and 22(12.22%) of the women accepted involvement. Postcoital bleeding, which may be a warning sign for cervical cancer was present in 4(2.22%) of the women.

In our study, 55(30.56%) of the women had knowledge of cervical cancer, however, only 28 of the 180 women (15.56%) have ever been screened for cervical cancer. This screened rate is about half of those among Jordanian women^3^, close to the 16.4% in Kenya^9^ but higher than 12.2% among Ethiopian women,^10^ and 4.8% in Eastern Uganda.^5^ A more recent study in Central Uganda had a screening uptake of 20.6%.^11^ The Kenyan study also showed that the screening rate was higher (25.2%) among educated women.^9^

A previous study in Nigeria had an uptake of 22.9%.^6^ However, a more recent study focused on the barriers and motivators for screening showed that though 41.4% of the women were aware of screening methods, only 18.4% had done a previous screen.^12^ This underscores the need for search for other drivers of screening uptake.

Previous studies have shown that the uptake of screening was better where women had adequate knowledge of the disease.^1^, ^11^ Even in an underserved Ugandan population, prompting by Health workers and having knowledge of the symptoms and signs of cervical cancer increased the uptake of screening.^5^

The theory of reasoned action (TRA) which holds that personal perception may influence actual behaviour, and the Health Belief Model (HBM), which holds that perception of the severity, susceptibility to illness and its consequences are key factors in predicting the likelihood to take a preventative action,^1^ may explain how knowledge of cervical cancer increases screening uptake. However, some researchers have reported that screening uptake was still low despite high perception of seriousness of the disease.^13^

In Figure 1, 94 (52.22%) of the women accepted to do Pap smear after counselling while 86 (47.78%) rejected the offer.

In Figure 2, two groups (Accept and Reject) were subjected to inferential statistics testing the effect of (knowledge of cervical cancer, previous screening, history of multiple sexual partners, and presence of offensive watery vaginal discharge), on acceptance to screen.

Our study showed that knowledge of cervical cancer significantly reduced the number of women who objected to screening (Figure 2A), and collaborates other studies earlier reported.

Women who have done a previous screen were not more likely to accept screening, as shown in Figure 2B. History of multiple sexual partners, and presence of offensive watery vaginal discharge also significantly reduced the number of women who rejected the offer to screen.

Offensive watery vaginal discharge is an often ignored and unrecognized early sign of cervical cancer. It becomes a recognized sign at the advanced stage when it has become profound and part of palliative care.^14^ It was present in 13(7.22%) of the women in this study, and a significant driver for the acceptance to screen.

## Conclusion

The drivers for cervical cancer screening from this study included knowledge of cervical cancer, positive history of multiple sexual partners, and presence of offensive watery vaginal discharge. Previously screened women were not more likely to accept screening.

## Recommendation

These drivers if targeted for behavioural change and health promotion are likely to increase uptake of cervical cancer screening services.
